# S-Adenosylmethionine Inhibits Plasminogen-Activating Inhibitor-1 and Protects Male Mice from FOLFOX-Induced Liver Injury

**DOI:** 10.1016/j.jcmgh.2025.101513

**Published:** 2025-04-17

**Authors:** Alexandra Gangi, Tony W.H. Li, Youngyi Lim, Swati Chandla, Andrea Floris, Arash Khangholi, Maria Lauda Tomasi, Shelly C. Lu

**Affiliations:** 1 Department of Surgery, Cedars-Sinai Medical Center, Los Angeles, California; 2 Karsh Division of Gastroenterology and Hepatology, Department of Medicine, Cedars-Sinai Medical Center, Los Angeles, California

**Keywords:** Colorectal Liver Metastasis, NF-κB, Sinusoidal Obstruction Syndrome

## Abstract

**Background & Aims:**

FOLFOX, often used in patients with colorectal liver metastases, can cause sinusoidal obstruction syndrome (SOS) hindering subsequent treatment. S-adenosylmethionine (SAMe) is hepatoprotective and here we investigated whether it protects against FOLFOX-induced hepatotoxicity and defined the underlying mechanisms.

**Methods:**

A murine model of FOLFOX-induced SOS examined the effect of SAMe and plasminogen-activating inhibitor-1 (PAI-1). In vitro studies included primary mouse and human hepatocytes, Kupffer cells, hepatic stellate cells, and liver sinusoidal endothelial cells.

**Results:**

SAMe cotreatment completely blocked the induction of markers increased in FOLFOX-induced SOS and protected against liver injury. The most up-regulated gene was *Serpine1*, which encodes for PAI-1. SAMe blocked FOLFOX-induced expression and activation of nuclear factor (NF)-κB, which is known to activate *SERPINE1/Serpine1* promoters. Interestingly, FOLFOX failed to activate hepatic NF-κB or cause liver injury in *Serpine1* knockout male mice. Treatment of mouse hepatocytes with recombinant PAI-1 induced NF-κB activation; conditioned media from recombinant PAI-1 or interleukin-1β-treated hepatocytes, but not exosomes, increased the expression of proinflammatory cytokines and *Cd31* in Kupffer cells and liver sinusoidal endothelial cells, respectively, which were blocked by SAMe. FOLFOX and interleukin-1β induced interaction between PAI-1 with urokinase plasminogen activator receptor in mouse liver and hepatocytes, respectively, which was blocked by SAMe. Recombinant PAI-1 requires interaction with uPA for full activation of NF-κB in hepatocytes. Neutralizing antibody against PAI-1 blocked interleukin-1β-mediated p65/PAI-1 activation in hepatocytes.

**Conclusions:**

FOLFOX treatment increased hepatocyte PAI-1 expression and liver injury, which were not observed in germline PAI-1 deficiency. Hepatocytes secrete PAI-1 to exert autocrine and paracrine effects to activate Kupffer cells and liver sinusoidal endothelial cells. SAMe protects against FOLFOX-mediated liver injury in part by inhibiting NF-κB activation and PAI-1 induction.

**Summary**

FOLFOX regimen can cause liver injury that interferes with subsequent care of patients with colorectal liver metastasis. Here we investigated the effect of S-adenosylmethionine and found it to protect mice from FOLFOX-induced liver injury, likely by suppressing plasminogen-activating inhibitor-1 expression.

Colorectal cancer is consistently among 1 of 3 most commonly observed malignancies worldwide and the second leading cause of cancer-related death in the United States.^1^ In 2024, approximately 152,810 new cases of colorectal cancer are expected in the United States. Of these patients, about 20%–25% present with liver metastases (colorectal cancer liver metastasis [CRLM]) at diagnosis, and an additional 18%–25% develop liver metastases during their disease course.^2^^,^^3^ With more routine use of chemotherapy, impressive tumor responses resulting in significant tumor downstaging allowing for curative hepatic resection in eligible patients with aggressive metastatic disease is now possible. The most commonly used regimen consists of a thymidylate synthase inhibitor (5-fluorouracil or capecitabine) in combination with oxaliplatin (FOLFOX).^4^ Given the increased use and success of this regimen, there are increasing numbers of patients with CRLM who undergo liver resection after receiving multiple cycles of systemic chemotherapy, which causes toxicities in different organs. With respect to the liver, 5-FU is known to induce hepatic steatosis primarily through mitochondrial dysfunction.^5^^,^^6^ Oxaliplatin-based regimens are known to cause hepatic parenchymal damage by inducing sinusoidal obstruction syndrome (SOS).^7^ SOS is characterized by hepatocyte atrophy, hepatic sinusoidal dilation, perisinusoidal dilation, and nodular regenerative hyperplasia.^8^ The frequency of oxaliplatin-mediated SOS ranges from 19%–78% and 5-FU induced liver steatosis, 30%–47%.^9^, ^10^, ^11^ With the oxaliplatin-induced SOS leading to impaired liver function, patients with CRLM may become ineligible for major liver resection or further systemic therapy.^12^^,^^13^ Also, appropriately selected patients who had resectable disease and adequate preoperative liver function have higher surgical morbidity when treated with FOLFOX before resection.^9^ Prevention and treatment of SOS are critical to improve outcome of FOLFOX-treated patients with CRLM.

Although much has been reported on the pathogenesis of SOS, there are no preventive therapies available in patients with CRLM treated with FOLFOX. Here we examined the utility of S-adenosylmethionine (SAMe) in a murine model of FOLFOX-induced SOS.^14^ The rationale for examining SAMe in this model is that the liver is the major site of SAMe synthesis, where SAMe serves as a major precursor of glutathione, the key cellular antioxidant.^15^ Administration of SAMe to patients with chronic liver disease raised hepatic glutathione levels.^16^ In addition, SAMe has multiple actions including preventing vascular endothelial cell dysfunction induced by high-fat diet^17^; inhibiting liver fibrosis in part via blocking transforming growth factor (TGF)-β-induced collagen synthesis^18^^,^^19^; and inhibiting release of proinflammatory cytokines, such as tumor necrosis factor-α (TNF-α).^20^ In this study, we discovered that SAMe protected against the development of FOLFOX-induced SOS in the murine model. We further identified the key target is plasminogen-activating inhibitor-1 (PAI-1), which is the most up-regulated marker in the FOLFOX-induced SOS model and is required for nuclear factor-κB (NF-κB) activation in hepatocytes. Consistently, PAI-1 knockout (KO) mice are resistant to FOLFOX-induced SOS, NF-κB activation, and liver injury. We elucidated the underlying molecular mechanism of how SAMe inhibits PAI-1 expression and how PAI-1 activates hepatocyte NF-κB in hepatocytes. Our findings have important implications in protecting patients with CRLM from FOLFOX-induced liver injury.

## Results

### SAMe Protects Against FOLFOX-Induced SOS and Liver Injury in Mice

**Figure 1:**
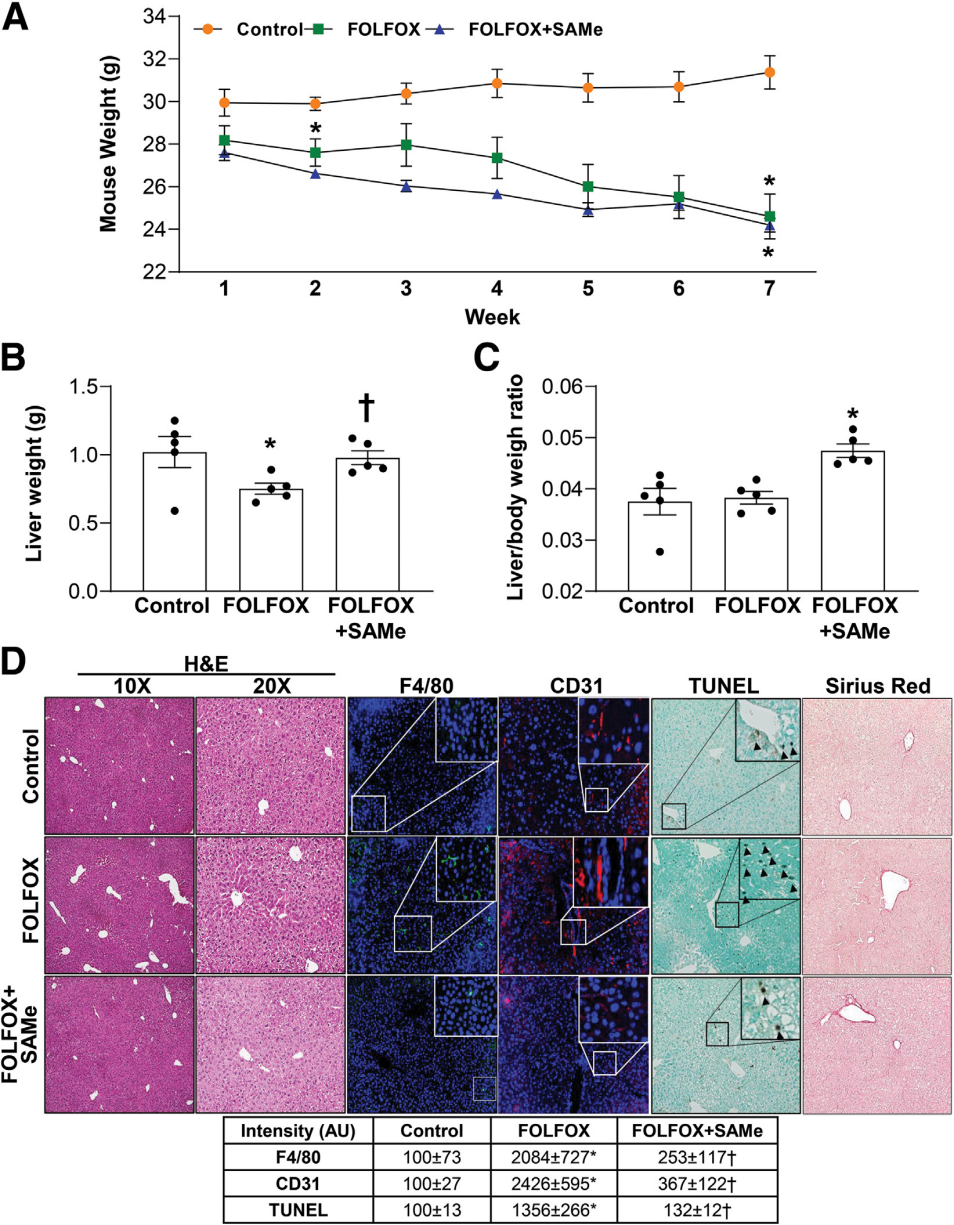
**SAMe inhibits FOLFOX-induced SOS, increased macrophage accumulation, and apoptosis in mouse liver.** Mice were treated with FOLFOX and vehicle or SAMe, whereas control animals received vehicles only as described in Methods and the following parameters were examined. (*A*) Body, (*B*) liver weight loss, and (*C*) liver/body weight ratios in C57BL/6J mice control or treated with FOLFOX ± SAMe as described in Methods. Values are expressed as mean ± standard error of the mean (n = 5). ∗*P* < .05 versus control. †*P* < .05 versus FOLFOX for body weight. ∗*P* < .001 versus control for liver/body weight. (*D*) Histology and immunohistochemistry of mouse liver tissues. Hematoxylin-eosin (under x100 and x200 magnifications), F4/80 immunofluorescence, CD31 TUNEL, and Sirius red staining of liver sections. *Boxed areas* are magnified further. *Arrowheads* point to TUNEL+ staining. Fluorescence and IHC staining intensity were quantified by ImageJ and summarized in the box below. Values of % intensity (AU) are expressed as mean ± standard error of the mean (n = 4). ∗*P* < .03 versus control, †*P* < .05 versus FOLFOX for F4/80. ∗*P* < .01 versus control, †*P* < .015 versus FOLFOX for CD31. ∗*P* < .003 versus control, †*P* < .003 versus FOLFOX for TUNEL.

**Figure 2:**
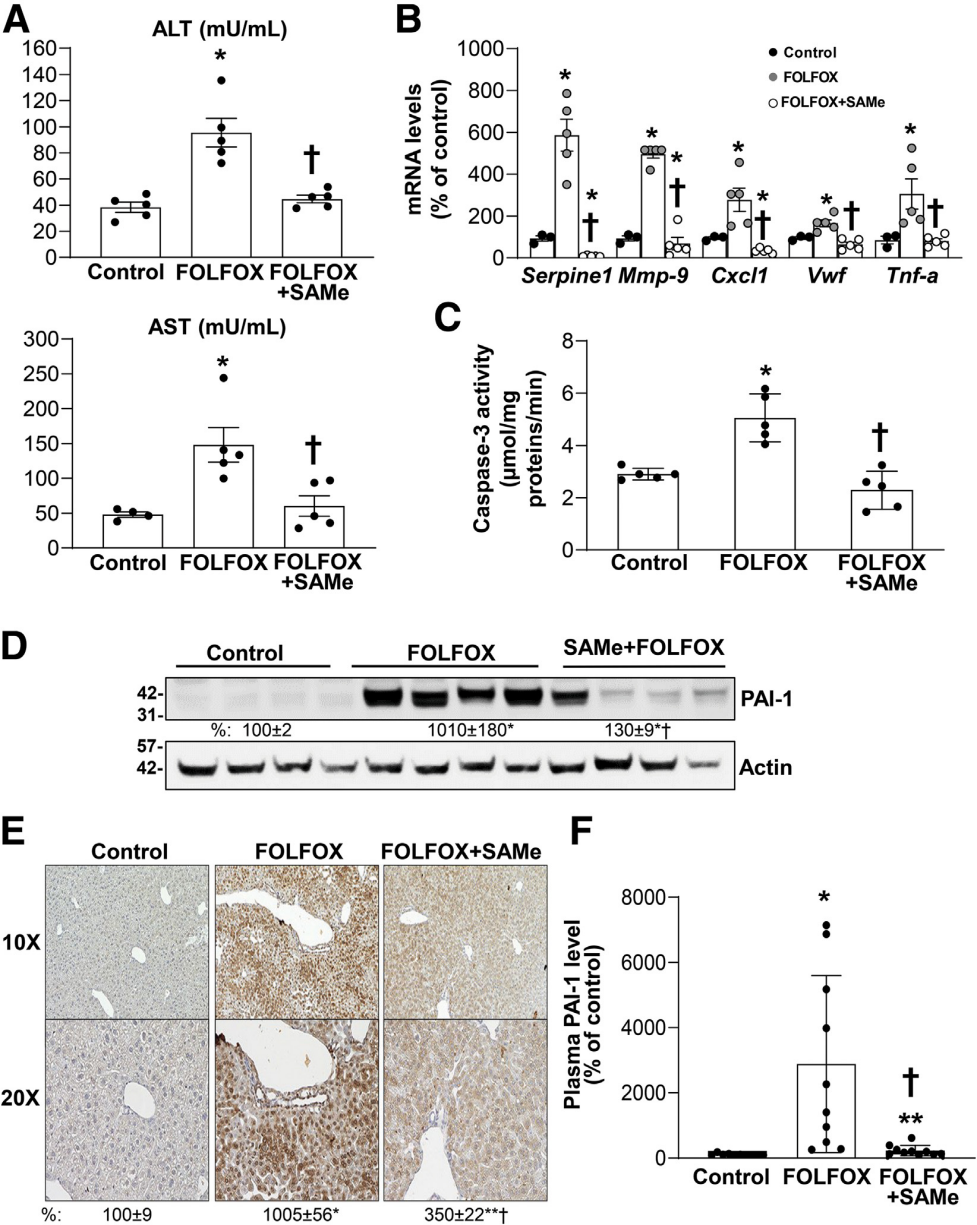
**SAMe inhibits FOLFOX-induced liver injury in mouse liver and PAI-1 induction.** Mice were treated with FOLFOX and vehicle or SAMe, whereas control animals received vehicles only as described in Methods and the following parameters were examined. (*A*) Plasma alanine aminotransferase (ALT) and aspartate aminotransferase (AST) levels were analyzed as indicated in Methods. Data represent mean ± standard error of the mean (n = 5). ∗*P* < .01 versus control, †*P* < .02 versus FOLFOX. (*B*) RNA was extracted from livers and expression of SOS gene markers (*Serpine1*, *Mmp9*, *Cxc1*, *Vwf*, *Tnf-α*) was analyzed by real-time polymerase chain reaction. Data represent mean ± standard error of the mean from n = 3–5. ∗*P* < .02 versus control, †*P* < .02 versus FOLFOX. (*C*) Colorimetric assay of caspase-3 activity in the livers of control mice or treated with FOLFOX ± SAMe for 5 weeks was measured as described in Methods. Values represent mean ± standard error of the mean from n = 5 per group. ∗*P* < .01 versus control, †*P* < .02 versus FOLFOX. (*D*) Protein lysates were extracted from livers to measure PAI-1 protein level by Western blotting. Densitometric ratios normalized to actin are summarized below the blot. Results are expressed as % of control (mean ± standard error of the mean) from 5 mice/group. ∗*P* < .001 versus control; †*P* < .04 versus FOLFOX. (*E*) Immunohistochemistry of PAI-1 expression in liver of FOLFOX-treated mice under low and high magnifications was quantified by ImageJ. Values represent mean ± standard error of the mean from n = 4 per group expressed as % of control. ∗*P* < .0001 and ∗∗*P* < .01 versus control, †*P* < .001 versus FOLFOX. (*F*) Plasma level of PAI-1 was measured by enzyme-linked immunosorbent assay as described in Methods. Data represent mean ± standard error of the mean from n = 10. ∗*P* < .004 versus control, †*P* < .01 versus FOLFOX.

Using a murine model of FOLFOX-induced SOS that resembles human SOS,^14^ we examine the effect of SAMe by treating C57BL/6 male mice with FOLFOX with or without SAMe. FOLFOX treatment led to a continuous weight loss, fall in liver weight, and although SAMe cotreatment had no effect on body weight, it prevented the fall in liver weight, and this resulted in a slight increase in liver to body weight ratio (Figure 1*A-C*). FOLFOX-treated livers exhibit sinusoidal dilatation consistent with SOS, accumulation of macrophages (F4/80+ cells), apoptosis (terminal deoxynucleotidyl transferase-mediated dUTP nick-end labeling [TUNEL] staining), increased CD31 staining (marker of capillarization),^21^ but no change in fibrosis; whereas SAMe cotreated mice were protected from all these changes (Figure 1*D*) and biochemical parameters of liver injury (Figure 2*A*). Several markers known to be increased in the murine model of FOLFOX-induced SOS,^14^ namely PAI-1 (encoded by *Serpine1*), TNF-α, matrix metalloproteinase-9 (MMP-9), CXCl1, and von Willebrand factor, were induced, with a 6-fold increase in *Serpine1* mRNA levels and SAMe cotreatment not only blocked their increase but the mRNA levels of some genes (*Serpine 1*, *Mmp-9*, *Cxcl1*) were even lower than baseline (Figure 2*B*). Consistent with higher TUNEL staining, caspase 3 activity is higher in FOLFOX-treated livers and this was blocked by SAMe cotreatment (Figure 2*C*). Because PAI-1 expression was the most induced and blocked by SAMe, we focused on PAI-1 in subsequent experiments.

### Mechanism of FOLFOX-Induced PAI-1 Expression and SAMe Inhibition

We first confirmed that PAI-1 expression is induced at the protein level (Figure 2*D*). In normal liver, PAI-1 is mainly expressed in liver sinusoidal endothelial cells (LSECs) but interestingly, PAI-1 induction after FOLFOX treatment occurs prominently in hepatocytes, and SAMe treatment strongly inhibited the induction in hepatocytes on immunohistochemistry (IHC) (Figure 2*E*). Plasma PAI-1 levels increased 20-fold after FOLFOX treatment, but this was completely blocked by SAMe cotreatment (Figure 2*F*).

**Figure 3:**
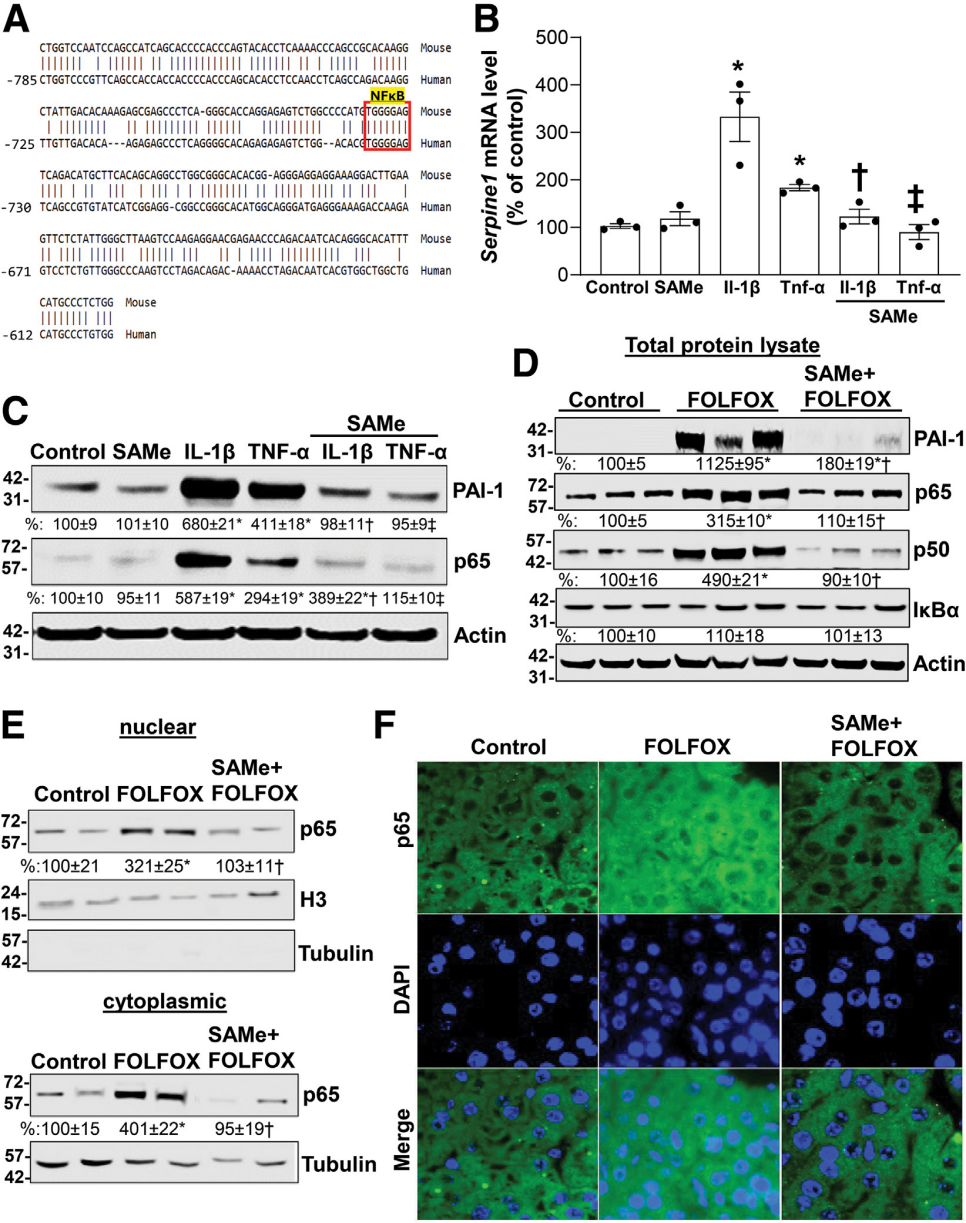
**SAMe treatment prevents FOLFOX, IL-1β-, and TNF-α-induced PAI-1 expression and p65 nuclear translocation.** Primary mouse hepatocytes (0.5 x 10^6^/6-well plate) were treated with 2 mM SAMe, 10 ng/mL IL-1β, 10 ng/mL TNF-α alone or in combination for 24 hours. (*A*) The mouse promoter was aligned to the human -785 bp promoter using BLAST tool. The identified NF-κB p65-binding site in both promoters is boxed (*red*). Total mRNA level of *Serpine1* was analyzed by real-time polymerase chain reaction (*B*), whereas (*C*) PAI-1 and p65 protein levels were analyzed by Western blotting. The densitometric ratios are represented as % of control and summarized below the blots. Data represent mean ± standard error of the mean from n = 3. ∗*P* < .01 versus control; †*P* < .01 versus IL-1β; ‡*P* < .02 versus TNF-α. (*D*) Protein lysates were extracted from livers of control and FOLFOX ± SAMe-treated mice to measure PAI-1, p65, p50, and IκBα protein levels by Western blotting. Densitometric ratios normalized to actin are indicated under each blot. Results are expressed as % of control (mean ± standard error of the mean) from 3 mice/group. ∗*P* < .02 versus control; †*P* < .01 versus FOLFOX. (*E*) Nuclear and cytoplasmic proteins were extracted from livers of FOLFOX ± SAMe-treated mice and analyzed by Western blotting, with densitometric values summarized below the blots, and (*F*) immunofluorescence to measure p65 protein level and subcellular localization (*green*), respectively. Cell nuclei were stained with DAPI (*blue*) (x400). Data represent mean ± standard error of the mean from n = 3. ∗*P* < .02 versus control and †*P* < .01 versus FOLFOX for nuclear fraction; ∗*P* < .01 versus control and †*P* < .05 versus FOLFOX for cytoplasmic fraction.

We next investigated how SAMe inhibits PAI-1 expression. PAI-1 is well known to be induced by treatments that activate NF-κB^22^ and there are consensus NF-κB binding sites in the promoter regions of the mouse and human PAI-1 promoters (Figure 3*A*). Because SAMe was shown to inhibit NF-κB activation,^20^ we examined whether this might be a mechanism for SAMe to inhibit PAI-1 expression. We treated primary cultures of mouse hepatocytes with TNF-α and interleukin (IL)-1β, which are known to induce PAI-I expression.^23^ Consistently, both TNF-α and IL-1β treatment induced the expression of PAI-1 and p65. SAMe treatment did not influence basal PAI-1 expression, but it blocked the increase in PAI-1 completely and p65 partially (Figure 3*B* and *C*). FOLFOX-treated livers also exhibited an induction in NF-κB family members (p65 and p50), which were blocked by SAMe cotreatment, whereas IκBα remained unchanged (Figure 3*D*). FOLFOX treatment resulted in higher hepatic nuclear and cytoplasmic p65 levels, which was blocked by SAMe on Western blot and immunofluorescence (Figure 3*E* and *F*).

**Figure 4:**
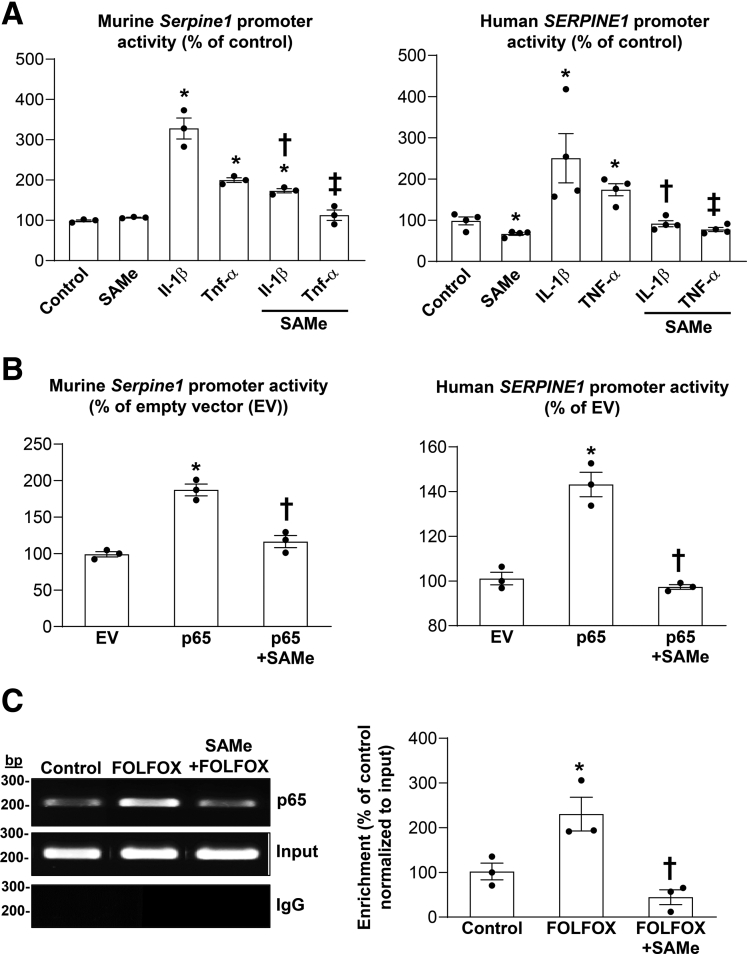
**SAMe administration lowers PAI-1 promoter activity in part by inhibiting p65 binding in vitro and in vivo.** (*A*) Primary human and mouse hepatocytes were transfected with human *SERPINE 1* and murine *Serpine 1* promoter-luciferase reporter plasmid, respectively, for 48 hours and treated with SAMe, IL-1β, TNF-α alone or combined for the last 24 hours as described in Methods. Relative Cypridina activity was normalized to Renilla luciferase activity. Data represent mean ± standard error of the mean. ∗*P* < .001 versus control, †*P* < .004 versus IL-1β, ‡*P* < .004 versus TNF-α for mouse hepatocytes (n = 3); ∗*P* < .04 versus control, †*P* < .04 versus IL-1β, ‡*P* < .001 versus TNF-α for human hepatocytes (n = 4). (*B*) Primary human and mouse hepatocytes were transfected with human *SERPINE 1* and murine *Serpine 1* promoter-luciferase reporter plasmid, respectively, and cotransfected with p65 overexpression or EV for 48 hours. SAMe treatment was added during the last 24 hours. Relative Cypridina activity was normalized to Renilla luciferase activity. Data represent mean ± standard error of the mean. ∗*P* < .001 versus EV, †*P* < .04 for mouse hepatocytes (n = 3); ∗*P* < .02 versus EV, †*P* < .001 for human hepatocytes (n = 3). (*C*) Chromatin immunoprecipitation showing direct binding of p65 to *Serpine 1* promoter region that contains the NF-κB element in the liver of FOLFOX-treated mice. P65 antibody (3 μg) was used. Chromatin immunoprecipitation DNA was amplified and resolved by 1% agarose gel. The genomic region between -785 and -612 was analyzed by Chromatin immunoprecipitation/polymerase chain reaction. Data represent mean ± standard error of the mean from n = 3. ∗*P* < .04 versus control, †*P* < .01 versus FOLFOX.

We next examined how these treatments affect the human and murine PAI-1 promoter activities using promoter constructs from Genecopoeia (Human *SERPINE1*, located in chr7+:101125713-101127383 [1671bp]; mouse *Serpine1*, located in chr5-:137073548-137071986 [1563bp]). We found IL-1β and TNF-α increased the murine and human PAI-1 promoter activities similar to mRNA levels and SAMe addition blocked the induction (Figure 4*A*). Overexpression of p65 also induced the human and murine PAI-1 promoter activities and this was blocked by SAMe (Figure 4*B*). In FOLFOX-treated livers, there is an increase in the binding of p65 to the *Serpine1* promoter region that has the NF-κB element, which was blocked by SAMe (Figure 4*C*).

### Role of PAI-1 in FOLFOX-Induced SOS in Mice

**Figure 5:**
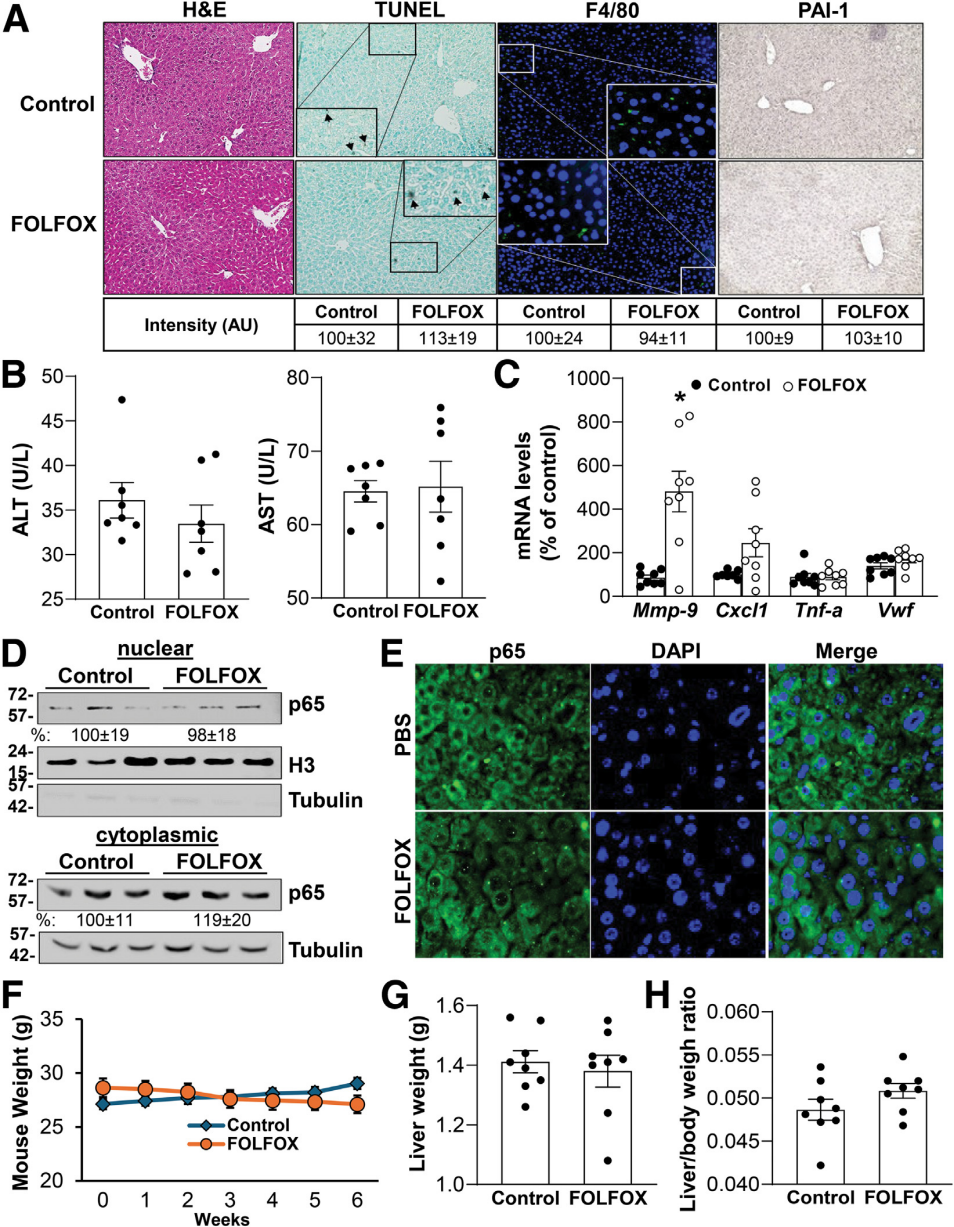
**Loss of PAI-1 protects from FOLFOX-induced liver damage in mice.** PAI-1 knockout mice were treated with FOLFOX as indicated in Methods. (*A*) Histology and immunohistochemistry of mouse liver tissues under magnification x200. Hematoxylin-eosin (H&E), F4/80 immunofluorescence, PAI-1, and TUNEL staining of liver sections. *Boxed areas* are further magnified. *Arrowheads* point to TUNEL+ staining. Fluorescence and IHC staining intensity were quantified by ImageJ and summarized in the box below. (*B*) Plasma alanine aminotransferase (ALT) and aspartate aminotransferase (AST) levels. Data represent mean ± standard error of the mean from n = 7. (*C*) RNA was extracted from livers and expression of SOS gene markers (*Mmp9*, *Cxc1*, *Vwf*, *Tnf-α*) was analyzed by real-time polymerase chain reaction. Data represent mean ± standard error of the mean from n = 8. ∗*P* < .01 versus control. (*D*) Nuclear and cytoplasmic proteins were extracted from livers of FOLFOX-treated mice and analyzed by Western blotting with densitometric values summarized below the blots, and (*E*) immunofluorescence to measure p65 protein level and subcellular localization (*green*), respectively. Cell nuclei were stained with DAPI (*blue*) (x400). Data represent mean ± standard error of the mean from n = 3. (*F*) Body, (*G*) liver weight loss, and (*H*) liver/body weight ratios in PAI-1 KO mice control or treated with FOLFOX for 5 weeks. Values are expressed as mean ± standard error of the mean from n = 8 per group.

We next investigated the role of PAI-1 in FOLFOX-induced liver injury. We found PAI-1 KO male mice were resistant to FOLFOX-induced SOS histologically and liver injury biochemically (Figure 5*A* and *B*). Of the 5 SOS markers, the only 1 induced was MMP-9 (in fact, induced at a higher level than in wild-type mice) (Figures 2*B* and 5*C*). In PAI-1 KO mice, FOLFOX treatment had no effect on NF-κB content on Western blot of nuclear and cytosolic fractions or on immunofluorescence microscopy (Figure 5*D* and *E*), suggesting PAI-1 induction drives NF-κB expression and activation. Interestingly, there was no effect of FOLFOX on body or liver weights (Figure 5*F-H*).

### PAI-1 Directly Activates NF-κB in Cultured Mouse Hepatocytes and Conditioned Media from These Hepatocytes Induce Activation of LSECs and Kupffer Cells

**Figure 6:**
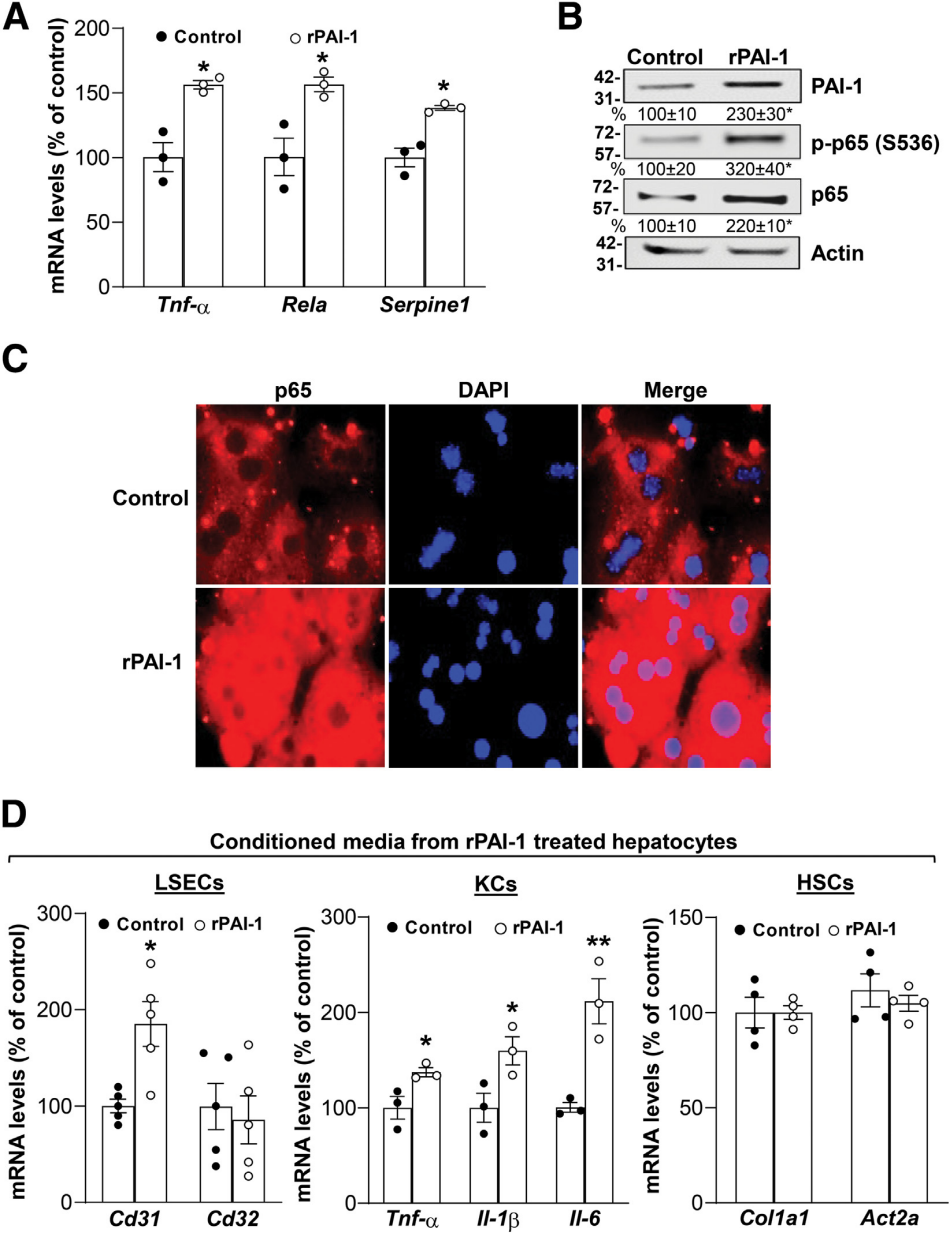
**PAI-1 activates NF-κB in mouse hepatocytes and causes hepatocytes to release factors that induce CD31 expression in LSECs and inflammatory cytokines in KCs.** Primary mouse hepatocytes were treated with 100 nM rPAI-1 for 24 hours. (*A*) mRNA levels of *Serpine 1*, *Rela*, and *Tnf-α* were measured by real-time polymerase chain reaction in primary mouse hepatocytes. Data represent mean ± standard error of the mean from n = 3. ∗*P* < .01 versus control. (*B*) Protein levels of PAI-1, p-p65 (S536), and p65 were analyzed by Western blotting. Actin was used as housekeeping. Densitometric values are summarized below the blots. Data represent mean ± standard error of the mean from n = 3. ∗*P* < .02 versus control. (*C*) Immunofluorescence of p65 (*red*). Cell nuclei were stained with DAPI (*blue*) (x400). (*D*) LSECs, KCs, and HSCs were cultured for 24 hours with conditioned media from rPAI-1-treated primary mouse hepatocytes. RNA was extracted to measure the gene expression of *Cd31* and *Cd32* in LSECs, *Tnf-α*, IL-1β, and IL-6 in KCs, and *Col I* and *Acta2* in HSCs by real-time polymerase chain reaction. Data represent mean ± standard error of the mean. ∗*P* < .01 versus control in LSECs (n = 5); ∗*P* < .04 and ∗∗*P* < .01 versus control in KCs (n = 3); HSCs (n = 4).

Intrigued by the data that PAI-1 seems to be required for NF-κB induction by FOLFOX and the observation that the this occurred prominently in hepatocytes, we treated primary mouse hepatocytes with active rPAI-1 (10 ng/mL) and found a significant increase in p65 expression and activity, higher *Tnf-α* and *Serpine1* mRNA levels (Figure 6*A-C*). Conditioned media from rPAI-1-treated hepatocytes also induced the expression of CD31 in LSECs and proinflammatory cytokines TNF-α, IL-1β, and IL-6 in Kupffer cells (KCs) (Figure 6*D*), whereas it had no influence on hepatic stellate cells (HSCs) activation markers *Col I* and *Acta2* (Figure 6*D*).

### FOLFOX Induces Interaction of PAI-1 with Vitronectin and Urokinase Plasminogen Activator Receptor, Which Was Blocked by SAMe, and the uPAR Signaling is Likely Required to Activate NF-κB

**Figure 7:**
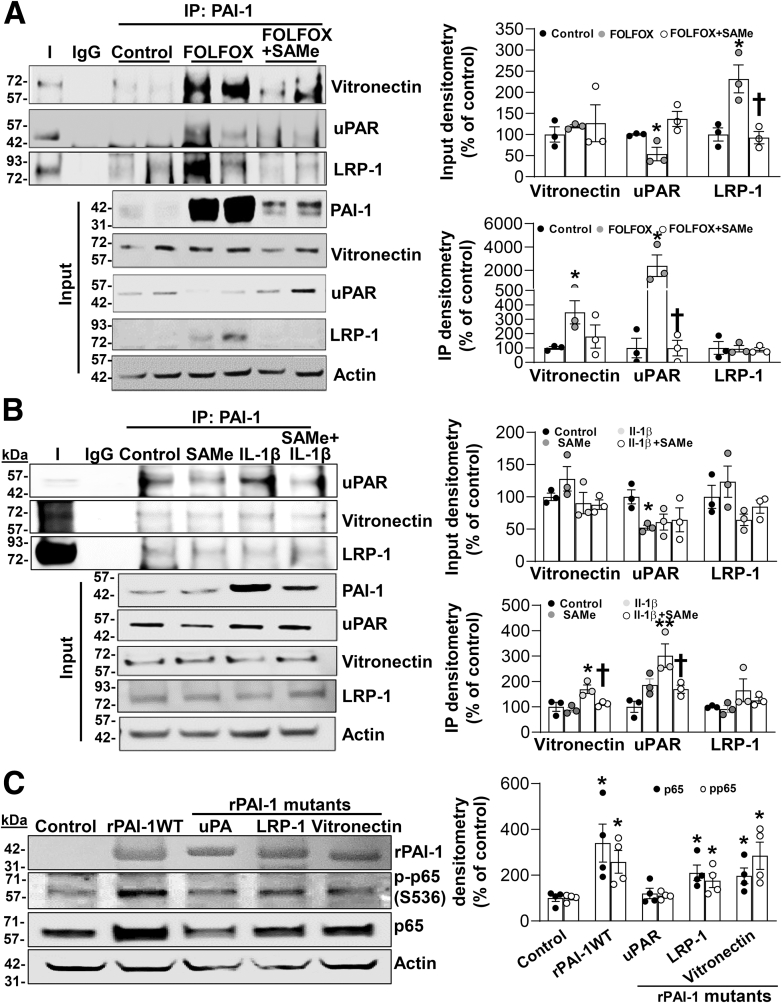
**SAMe treatment inhibits FOLFOX and IL1β-induced PAI-1/uPAR complex formation, which is required for NF-κB activation.** (*A*) Protein lysates from FOLFOX ± SAMe-treated and control mouse livers were used to immunoprecipitate (IP) PAI-1 and analyzed for complex formation with uPAR, LRP-1, and vitronectin with Western blotting. Actin was used as housekeeping. Graphs on the right summarize densitometric changes of vitronectin, uPAR, and LRP-1 in response to the treatments (*top*) and bound vitronectin, uPAR, and LRP-1 to immunoprecipitated PAI-1 (*bottom*) in liver lysates. Data represent mean ± standard error of the mean from n = 3. ∗*P* < .04 versus control, †*P* < .02 versus FOLFOX for immunoblotting (IB); ∗*P* < .03 versus control, †*P* < .04 versus FOLFOX for IP. (*B*) Primary mouse hepatocytes were treated with 2 mM SAMe, 10 ng/mL IL-1β, alone or together for 24 hours. Cells were lysed and proteins extracted to analyze PAI-1 coimmunoprecipitation with uPAR, LRP-1, and vitronection by Western blotting. Graphs on the *right* summarize densitometric changes of vitronectin, uPAR and LRP-1 in response to the treatments (*top*) and bound vitronectin, uPAR and LRP-1 to immunoprecipitated PAI-1 (*bottom*) in cell lysates. Data represent mean ± standard error of the mean from n = 3. ∗*P* < .01 versus control for IB; ∗*P* < .04 and ∗∗*P* < .01 versus control, †*P* < .05 versus IL-1β for IP. (*C*) Primary mouse hepatocytes were treated with rPAI-1 wild-type and mutants that cannot bind uPA, LRP-1, and vitronectin (100nM) for 24 hours. Protein levels of PAI-1, p-p65 (S536), and p65 were analyzed by Western blotting with actin as housekeeping control and densitometric values are summarized in the graph on the *right*. Data represent mean ± standard error of the mean from n = 4. ∗*P* < .04 versus control.

Much of PAI-1’s effect is exerted via its ability to interact with multiple proteins in the extracellular matrix (ECM) to influence their signaling. We next investigated the effect of FOLFOX and SAMe on PAI-1’s interaction with 3 well-known proteins: urokinase plasminogen activator receptor (uPAR), vitronectin, and low-density lipoprotein receptor-related protein-1 (LRP-1). We found FOLFOX treatment induced the expression of LRP-1, lowered the expression of uPAR, but did not alter the expression of vitronectin. However, interaction between PAI-1 and vitronectin or uPAR was markedly induced after FOLFOX, but not that of the interaction between PAI-1 with LRP-1 (Figure 7*A*). SAMe cotreatment blocked the increase in LRP-1 expression, and increased interaction of PAI-1 with uPAR (Figure 7*A*). In mouse hepatocytes treated with IL-1β, there is also an increase in the interaction between PAI-1 and uPAR and vitronectin, which was blocked by SAMe (Figure 7*B*). Expression of LRP-1 was unchanged and interaction between PAI1 and LRP-1 was also unchanged after IL-1β treatment (Figure 7*B*).

To better understand the signaling pathways that are required for PAI-1 to activate NF-κB in hepatocytes, we used rPAI-1 with different mutations that prohibit interaction with LRP-1 (R76E/I91L), urokinase-type plasminogen activator (uPA; T333R/A335R), or vitronectin (K154T/Q319L/M354I/N150H). Because recombinant PAI-1 uPAR binding negative mutant is not commercially available, we used the uPA binding negative mutant. We found interaction with uPA (but not vitronectin or LRP-1) is required to activate NF-κB, because the PAI-1 mutant unable to interact with uPA could not activate NF-κB (Figure 7*C*).

### Hepatocytes Secrete PAI-1 Freely and in Exosomes, Which Activates KCs NF-κB and LSECs CD31 Expression

**Figure 8:**
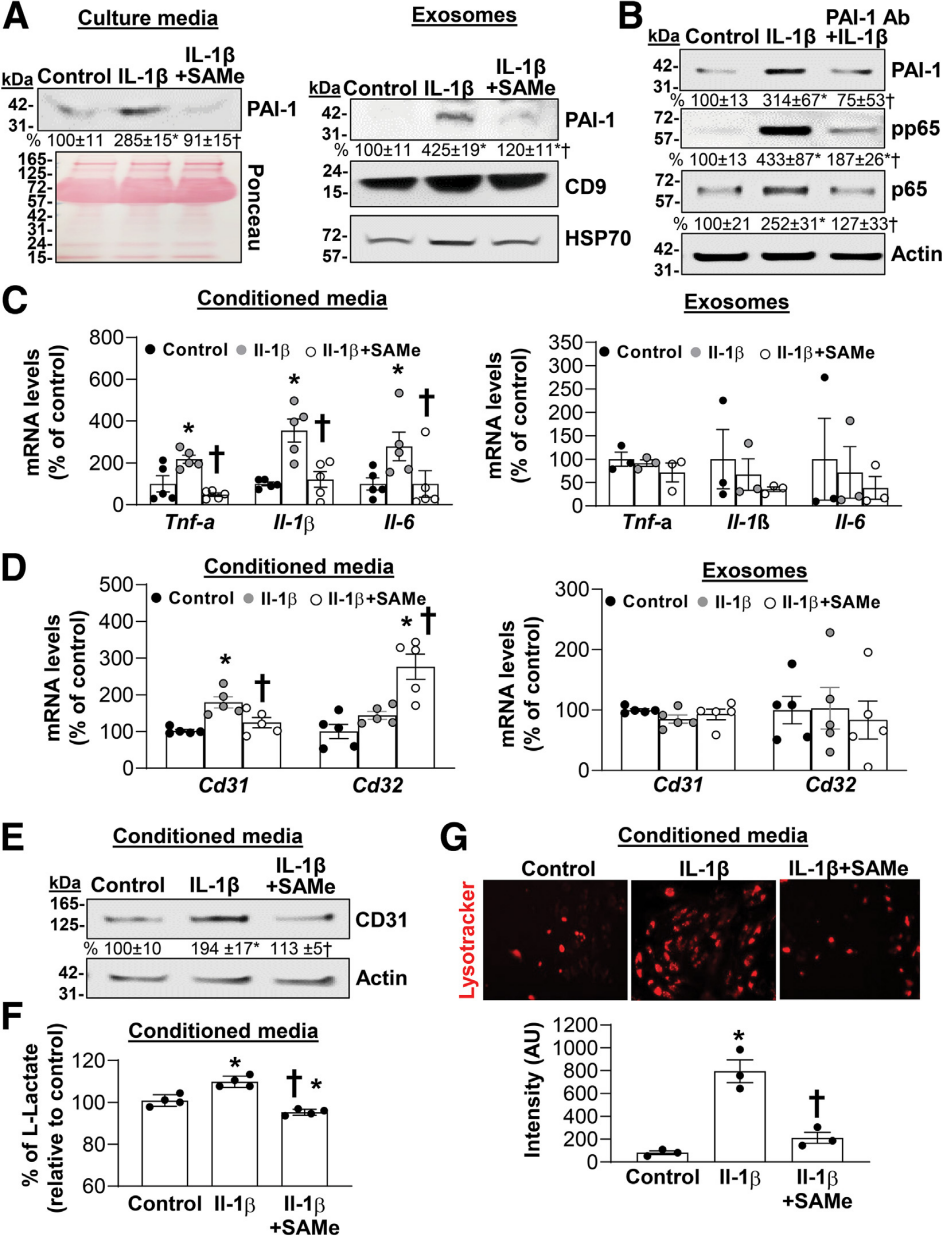
**SAMe inhibits PAI-1 secretion from hepatocytes and prevents LSECs and KCs activation.** Primary mouse hepatocytes were treated with 2 mM SAMe, 10 ng/mL IL-1β, alone or together for 24 hours. (*A*) PAI-1 protein level from culture media and exosomes was analyzed by Western blotting. Ponceau staining was used as loading control for culture media; CD9 and heat shock protein 70 were used as housekeeping for exosomes. Densitometric values are shown below the blots. Data represent mean ± standard error of the mean from n = 3. ∗*P* < .02 versus control and †*P* < .03 versus IL-1β; ∗*P* < .01 versus control and †*P* < .04 versus IL-1β exosomes. (*B*) Hepatocytes were treated with IL1β alone or with neutralizing antibody against PAI-1 (25 μg/mL) for 24 hours. Proteins lysates were analyzed to measure PAI-1, p65, and pp65 protein levels by Western blotting. Actin was used as housekeeping. Densitometric values are shown below the blots. Data represent mean ± standard error of the mean from n = 3. ∗*P* < .05 versus control, and †*P* < .05 versus IL-1β. (*C*) KCs and (*D*) LSECs cells were cultured with conditioned media or exosomes from mouse hepatocytes treated as described previously and RNA was extracted. mRNA levels of *Cd31* and *Cd32* in LSECs, *Tnf-α*, *Il-1β*, and *Il-6* in KCs were measured by real-time polymerase chain reaction. Data represent mean ± standard error of the mean from n = 3–5. ∗*P* < .03 versus control and †*P* < .01 versus IL-1β in conditioned media-treated KCs; ∗*P* < .004 versus control and †*P* < .04 versus IL-1β in conditioned media-treated LSECs. (*E*) CD31 protein level from LSECs treated as described previously. Results mean ± standard error of the mean from n = 3. ∗*P* < .04 versus control and †*P* < .05 versus IL-1β. (*F, G*) Glycolysis and lysosomal activity were measured by L-Lactate (mM) assay and lysotracker staining in LSECs cultured with conditioned media from mouse hepatocytes treated as indicated. Fluorescence intensity was quantified by ImageJ. Data represent mean ± standard error of the mean. ∗*P* < .03 versus control and †*P* < .001 versus IL-1β for L-Lactate level (n = 4). ∗*P* < .02 versus control and †*P* < .01 versus IL-1β for Lysotracker intensity (n = 3).

PAI-1 is secreted in the free form and within empty vectors.^24^^,^^25^ Liver has been shown to secrete PAI-1 in vivo and in vitro under normal physiological condition.^26^ We found in mouse hepatocytes SAMe lowered PAI-1 content in both forms and blocked the increase induced by IL-1β (Figure 8*A*). Intriguingly, addition of neutralizing antibody against the free form of PAI-1 in culture media prevented/attenuated the IL-1β-mediated increase of p65 expression and activity, and blocked PAI-1 induction completely in mouse hepatocytes (Figure 8*B*), suggesting freely secreted PAI-1 acts in an autocrine manner to induce its own expression. We also found that conditioned media, but not exosomes, from hepatocytes treated with IL-1β induced the expression of proinflammatory cytokines TNF-α, IL-1β, and IL-6 in KCs and CD31 expression in LSECs, and all were blocked if hepatocytes were also treated with SAMe except for CD32, which was increased above baseline (Figure 8*C-E*). On injury, LSECs become profoundly deregulated and are characterized by increased level of glycolysis and lysosomal activity.^27^^,^^28^ Consistently, LSECs treated with conditioned media from IL-1β-treated hepatocytes have higher L-lactate levels (product of glycolysis) and lysosomal activity (Figure 8*F* and *G*).^29^ These were not observed when LSECs were treated with conditioned media from hepatocytes treated with IL-1βplus SAMe (Figure 8 *F* and *G*).

## Discussion

The goal of this project was to investigate whether SAMe administration can prevent FOLFOX-induced liver injury. This was prompted by the unmet clinical need that many patients with CRLM become ineligible for the planned surgery because of chemotherapy-induced liver injury.^9^ In the course of our study, we found SAMe to be an excellent candidate to prevent FOLFOX-induced liver injury and identified a major role that PAI-1 plays in the pathogenesis of the injury.

SAMe is widely available in the United States as a health supplement. SAMe is the principal methyl donor and the liver is the body’s SAMe factory because half of our daily intake of methionine is catabolized to SAMe in the liver.^15^ In the liver SAMe is also a major precursor for glutathione through the transsulfuration pathway.^30^ SAMe has multiple pharmacologic actions: (1) hepatoprotective but proapoptotic in liver cancer^31^ and colon cancer cells,^32^ (2) prevent vascular endothelial cell dysfunction,^17^ (3) inhibit liver fibrosis,^18^ (4) inhibit release of proinflammatory cytokines by activated KCs,^20^ and (5) inhibit CRLM in a mouse model.^33^ Indeed, we found SAMe administration completely protected against FOLFOX-induced liver injury in mice. Consistently, a retrospective study of patients with colorectal cancer treated with FOLFOX found patients taking SAMe supplement experienced less liver injury and had a reduced need of course delay or dose reduction.^34^ However, a prospective randomized placebo-controlled clinical trial has not been reported and our results would support such a trial, which is being planned.

Although SOS has been widely recognized as a complication of oxaliplatin-based chemotherapy, other etiologies have been reported, including exposure to pyrrolizidine alkaloids found in certain herbal medicines, such as *Gynura segetum*, *Crotalaria*, and Senecio species, which undergo metabolic activation in the liver. Thus, understanding these alternative causes is essential for early diagnosis and management of SOS development.^35^ The pathogenesis of SOS involves at least 2 distinct and interrelated pathways. There is initial injury to SECs causing hepatic venous obstruction and congestion and downstream hepatic necrosis. Concurrently, sinusoidal endothelial cell injury leads to secretion of proinflammatory cytokines, which activate KCs and HSCs.^36^ The molecular pathophysiology of oxaliplatin-induced SOS involves the depolymerization of F-actin in LSECs, which results in increased expression of MMP-9 and MMP-2, which are thought to facilitate development of SOS by breaking down the ECM.^37^ However, it should be noted that PAI-1 KO mice treated with FOLFOX were completely resistant to the development of SOS despite a marked elevation in MMP-9 (in fact, the only marker that remained elevated after FOLFOX treatment), suggesting other mechanisms are more important in FOLFOX-induced SOS. Although abundant literature exists on the role of LSECs in SOS, much less is known about the role of hepatocytes.

We used the murine model of FOLFOX-induced SOS because it recapitulated the human pathology,^14^^,^^38^ and focused on *Serpine1* (encodes for PAI-1) because this was the most up-regulated marker that was blocked by SAMe. PAI-1 belongs to the serpin family and is an essential regulator of fibrinolysis by inhibiting the activity of tissue-type plasminogen activator and uPA.^23^ Tissue-type plasminogen activator binds to fibrin, converts plasminogen to plasmin in the clot, and is the primary fibrinolytic activator; whereas uPA has low affinity for fibrin but instead is the major plasminogen activator expressed by migrating cells and its plasmin activating activity is controlled by binding to its receptor uPAR.^39^ Plasmin can degrade many components of the ECM, and activate MMPs and growth factors, such as TGF-β and vascular endothelial growth factor.^39^ By inhibiting the plasminogen activator system, PAI-1 can influence many processes indirectly. However, PAI-1 also interacts with other ligands, such as heparin; ECM component vitronectin; and scavenger receptors, such as LRP-1. Many of its effects are related to these interactions.^39^ Binding to vitronectin stabilizes PAI-1 and interferes with binding of vitronectin to avb3 integrin and uPAR; whereas binding to uPAR makes PAI-1 preferentially bind to LRP-1 and the uPA/uPAR/PAI-1 complex is endocytosed for degradation of uPA and PAI-1 while uPAR is recycled.^40^ We found FOLFOX markedly increased interaction of PAI-1 with uPAR, which was blocked by SAMe cotreatment. Interaction between PAI-1 and vitronectin was also higher in FOLFOX-treated livers and IL-1β-treated hepatocytes but to a much lower magnitude as compared with uPAR and SAMe did not exert a statistically significant effect on their interaction in FOLFOX-treated livers. Increased interaction with vitronectin likely also contributed to the high PAI-1 expression because this is known to stabilize PAI-1.

In normal liver, PAI-1 is mainly expressed in LSECs but interestingly, PAI-1 induction after FOLFOX treatment occurred prominently in hepatocytes, and SAMe treatment strongly inhibited the induction in hepatocytes on IHC. In obesity, PAI-1 has also been reported to be induced in hepatocytes.^41^ Plasma PAI-1 levels increased 20-fold after FOLFOX treatment. Elevated plasma PAI-1 levels have been linked to many diseases, including cardiovascular disease, metabolic syndrome including metabolic-dysfunction-associated steatotic liver disease (previously known as nonalcoholic fatty liver disease), fibrosis, inflammation, neurodegenerative diseases, and cancer.^40^ PAI-1 is mainly produced by platelets and the endothelium but also expressed in many other cell types including adipocytes and macrophages.^23^^,^^39^ Its expression is induced by many cytokines (eg, TNF-α, IL-1β) and growth factors (eg, TGF-β, epidermal growth factor); however, there is a positive reciprocal regulation between PAI-1 and proinflammatory cytokines and TGF-β.^23^^,^^42^ PAI-1 is induced in human SOS and has been used as a biomarker for SOS.^38^^,^^43^^,^^44^ Elevated plasma levels and hepatic expression of PAI-1 correlate with degree of liver fibrosis and steatosis, whereas PAI-1 deletion or inhibition can ameliorate liver fibrosis and block high-fat diet-induced metabolic-dysfunction-associated steatotic liver disease.^45^, ^46^, ^47^ Supporting a causative role of PAI-1, PAI-1 KO mice are protected from FOLFOX-induced SOS. However, because we used PAI-1 KO mice that have total body PAI-1 deletion, it remains to be confirmed with hepatocyte-specific PAI-1 KO, which is planned for the future. PAI-1 is well known to be induced by treatments that activate NF-κB,^22^ and we found a key mechanism for SAMe to inhibit PAI-1 expression is via its ability to inhibit NF-κB activation. However, because the magnitude of induction in *SERPINE1/Serpine1* promoter activities was higher in response to IL-1β than overexpressing p65, there are also NF-κB-independent mechanisms. In addition, the magnitude of induction at the protein level was much higher than at the mRNA level of PAI-1, suggesting posttranslational regulation is also involved. One such mechanism is increased interaction with vitronectin, which is known to stabilize PAI-1,^39^ but there may be others that will require further study.

Although PAI-1 is widely known to be activated by NF-κB, we found that PAI-1 is required for FOLFOX to induce NF-κB. Ability of PAI-1 to activate NF-κB directly was confirmed in mouse hepatocytes treated with active rPAI-1. Our rPAI-1 dose is within normal plasma level, which reflects a combination of free, latent, and inactive forms, which can increase >10-fold in patients with SOS.^48^ We speculate the reason we observed such a robust response with 10 ng/mL in mouse hepatocytes is because the recombinant form we used is fully active. In FOLFOX-treated livers, increased interaction of PAI-1 with uPAR and vitronectin may contribute to NF-κB activation. In 1 study, high PAI-1 expression was found to activate p38MAPK with subsequent activation of p65-STAT3-IL-6 cascade, which can activate NF-κB, in macrophages and this action required the uPA binding domain of PAI-1.^49^ Vitronectin has also been reported to activate NF-κB through interacting with av integrins.^50^ However, how does PAI-1 activate NF-κB, because hepatocytes including hepatoma cells reportedly do not express uPAR?^51^ Contrary to previous report, we found hepatocytes express uPAR, and vitronectin and LRP-1. IL-1β treatment increased the interaction between PAI-1 and uPAR and vitronectin in mouse hepatocytes, which was blocked by SAMe. Taken together, the heightened interaction between PAI-1 and uPAR/vitronectin in mouse hepatocytes is the likely mechanism for NF-κB activation by PAI-1.

Lastly, PAI-1 is released as a free form and within exosomes.^24^ We found conditioned media but not exosomes from hepatocytes treated with rPAI-1 was able to activate proinflammatory cytokines from KCs and increase CD31 expression in LSECs, suggesting the importance of hepatocytes’ PAI-1 induction and release in contributing to the proinflammatory state and perpetuating the liver injury. Results with neutralizing antibody to PAI-1 of conditioned media confirmed the freely secreted PAI-1 by hepatocytes can also exert an autocrine effect to activate its own expression.

In summary, PAI-1 seems to be required for FOLFOX-mediated NF-κB activation, which then further induces PAI-1 expression in hepatocytes in a feedforward manner to cause liver injury by releasing PAI-1 to exert autocrine and paracrine effects. SAMe protects against FOLFOX-mediated liver injury in a large part by inhibiting NF-κB activation and PAI-1 induction. Given its well-established safety profile and availability as a dietary supplement in the United States, SAMe could be integrated into clinical practice as a prophylactic or adjunctive therapy in patients with CRLM receiving FOLFOX chemotherapy to protect against liver injury.

## Methods

### Materials and Reagents

SAMe, in the stable form of disulfate p-toluene sulfonate dried powder, was generously provided by Gnosis SRL (Cairate, Italy) and FOLFOX was purchased from Sigma-Aldrich (St. Louis, MO). Recombinant human PAI-1 wild-type (Cat.# IHUPAI1RSM) and mutants (R76E/I91L: cat.# IHUPAI1RLRP; T333R/A335R: Cat.# IHUPAI1RP12P14SB; K154T/Q319L/M354I/N150H: Cat.# IHUPAI1RSMVN) were purchased from Innovative Research (Novi, MI), whereas TNF-α (cat. # RTNFAI) and IL-1β (cat.# a42509) were purchased from Invitrogen (Waltham, MA). Table 1 provides a list of the antibodies used.

**Table 1:** List of Antibodies

Antibody	Catalog #	Manufacturer
PAI-1	ab182973	Abcam
	(WB, IP, IHC)	
	gtx89568	Genetex
	(neutralizing antibody)	
Vitronectin	15833-1-AP	Proteintech
p65	ab16502	Abcam
Tubulin	hrp66031	Proteintech
H3	ab21054	Abcam
Actin	a3854	Sigma
uPAR	10286-1-AP	Proteintech
LRP-1	NBP2-62753	NovusBio
p-p-65	3033S	Cell Signaling
CD9	20597-1-AP	Proteintech
HSP70	10995-1-AP	Proteintech
CD31	ab9498	Abcam
	(immunofluorescence)	
CD31	NB100-2284	Novus
	(western blot)	
F4/80	gr3263883-9	Sigma
IκBα	ab32518	Abcam

### Cell Culture and Treatments

Cryopreserved human hepatocytes were provided by Thermo Fisher (Waltham, MA). Briefly, frozen vial was rapidly thawed in a 37°C water bath and the content was mixed with 35 mL of seeding medium (Dulbecco's modified Eagles' medium [DMEM; Thermo Fisher], 10% fetal calf serum [Fisher Scientific], 1% penicillin/streptomycin solution [ScienCells]) and 15 mL of 90% Percoll solution (Sigma-Aldrich) in a 50-mL tube. Thereafter the tube was gently inverted 3 times and centrifuged at 96 x *g* for 6 minutes at room temperature. Next, the pellet was resuspended in 45 mL of seeding medium and centrifuged at 72 x *g* for 4 minutes at room temperature. Finally, the pellet was resuspended and cells were seeded as indicated for 4 hours before treatments.

Primary mouse hepatocytes, KCs, HSCs, and LSECs were simultaneously isolated from 3-month-old male C57BL/6 mice liver according to the methods described next. Mouse liver was perfused through the superior vena cava with Eagle's minimal essential medium at 5 mL/minute for 10 minutes, and then with digestion buffer (DMEM containing 0.044% [wt/vol] collagenase; Sigma) for 6–10 minutes. The liver was agitated in a rotary shaker for 10–15 minutes to further digest and dissociate the cells in DMEM containing 10 μg/mL DNase I (Sigma). The cell suspension was centrifuged at 700 rpm (50 × *g*) for 1 minute at 4°C. The resulting cell pellet was resuspended in 10 mL of Hanks buffer (Thermo Fisher) mixed with 5 mL Percoll solution (Sigma), and centrifuged at 150 × *g* for 5 minutes at 4°C. The cell pellet containing hepatocytes was washed twice with DMEM medium and centrifuged at 700 rpm (50 × *g*). The viability of hepatocytes was checked by trypan blue exclusion. Cells were plated at a density of 0.5 x 10^6^ cells per well in 6-well plates or 4 x 10^6^ cells per 10-cm/dish. Cultures were maintained in DMEM medium supplemented with 10% fetal calf serum, 2 mM glutamine, 50 mM penicillin, and 50 mg/mL streptomycin sulfate. The supernatant containing nonparenchymal cells was pelleted by centrifugation (800 × *g*, 10 minutes, 4°C) and resuspended in 8 mL 30% Percoll PLUS/Percoll (GE Healthcare Life Sciences, Freiburg, Germany), placed in 15-mL polystyrene conical centrifuge tube (BD Biosciences), and overlaid with 3 mL 70% Percoll solution.^52^ After being centrifuged at 2500 rpm for 20 minutes at room temperature with decreased acceleration and without breaks, the various cell types were arranged according to their density. HSCs were enriched in an upper cell layer, whereas KCs and LSECs fractions were floating between 50% and 60% Percoll. Cell fractions were collected separately by pipetting. The KCs/LSECs fraction was pelleted and KCs were labeled with CD14+MicroBeads (Miltenyi Biotec, Teterow, Germany) according to the manufacturer’s instructions. Cells were applied onto LS magnetic-activated cell sorting (MACS) columns (Miltenyi Biotec), which were placed within the magnetic field of a MACS separator and washed 3 times with MACS buffer (Miltenyi Biotec). CD14+KCs were eluted from the column by using 5 mL DMEM supplemented with 10% fetal bovine serum, 100 U/mL penicillin, 0.1 mg/mL streptomycin, and 2 mM L-glutamine. Viable KCs were counted and seeded onto plastic culture plates at a density of 6 x 10^5^ cells per 12-well/plate. Plates were gently washed 30 minutes after seeding and were then incubated at 37°C and 5% CO_2_. The flow-through collected during KCs separation was used to isolate LSECs. The LSECs were purified with a comparable MACS-based procedure using CD146+MicroBeads. Cells were eluted in endothelial growth medium (PromoCell, Heidelberg, Germany) containing provided supplements, 100 U/mL penicillin, and 0.1 mg/mL streptomycin and were then seeded 5 x 10^5^ cells per 12-well/plate coated with collagen-I.^53^

### Animal Study

Four-month-old male C57BL/6 and C57BL/6/SJL PAI-1 KO mice were purchased from the Jackson Laboratory (Bar Harbor, ME) and Innovative Research, respectively. Mice were fed, ad libitum, a synthetic irradiated standard diet from Research Diets, Inc (cat.# D01060501i; New Brunswick, NJ) and housed in a temperature-controlled animal facility with 12-hour light/dark cycles. All procedure protocols, use, and the care of the animals were reviewed and approved by the Institutional Animal Care and Use Committee at Cedars-Sinai Medical Center.

C57BL/6 mice were divided into 3 groups: control, FOLFOX treatment, and FOLFOX plus SAMe. C57BL/6/SJL PAI-1 KO mice were divided into 2 groups: control and FOLFOX treatment. The animals received weekly intraperitoneal injection of FOLFOX (6 mg/kg/body weight oxaliplatin, followed by an intraperitoneal injection of 50 mg/kg/body weight 5-FU and 90 mg/kg/body weight folinic acid) 2 hours later. Control animals were treated with vehicle alone. SAMe or phosphate-buffered saline 1X was administered via oral gavage daily, starting 3 days before the first FOLFOX treatment for the duration of the experiment (42 days) at 100 mg/kg/body weight. After 5 weeks of FOLFOX treatment, the mice were anesthetized and euthanized, the liver tissues were removed, snap frozen in liquid nitrogen, and stored at -80°C until analysis.

### RNA Extraction and Real-Time Polymerase Chain Reaction Analysis

Total RNA isolated from primary human, mouse hepatocytes, and mouse liver tissues as described^54^ was subjected to reverse transcription by using M-MLV Reverse Transcriptase (Invitrogen, Carlsbad, CA). One microliter of reverse transcription product was subjected to quantitative real-time polymerase chain reaction analysis. The primers and TaqMan probes for *Serpine1*, *Mmp9*, *Cxc1*, *Vwf*, *Tnf-α*, *IL-1β*, *Rela*, *Col-1*, *Acta2*, *SERPINE*, and Universal polymerase chain reaction Master Mix were purchased from ABI (Foster City, CA). *Gapdh* was used as housekeeping gene as described.^55^ The delta Ct (ΔCt) obtained was used to find the relative expression of genes according to the formula: relative expression = 2^-ΔΔCt^, where ΔΔCt = ΔCt of respective genes in experimental groups – ΔCt of the same genes in control group.

### Western Blots and Coimmunoprecipitation

Total protein lysates from primary human and mouse hepatocytes and mice livers were prepared as described^56^ and immunoprecipitated when indicated by specific PAI-1 antibody (cat.# ab222754; Abcam) and processes as reported.^57^ Immunoprecipitated proteins were subjected to Western blotting following standard protocols (Amersham BioSciences, Piscataway, NJ) and the membranes were probed with the following antibodies: PAI-1 (cat.# ab182973), p65 (cat.#ab16502), and IκBα (cat.# ab#32518) from Abcam; p-p65 (S536) (cat.# 3033S) from Cell Signaling (Danvers, MA); uPAR (cat.# 10286) and Vitronectin (cat.# 15833) from Proteintech (Rosemont, IL) and LRP-1 (cat.# NBP2-62753) from Novusbio (Centennial, CO); CD9 (cat. # 13174T) and heat shock protein 70 (cat.# 4872T) from Cell Signaling; and CD31 (cat.# ab9498) from Abcam. Nuclear and cytoplasmic proteins were prepared using the NE-PER Nuclear and Cytoplasmic Extraction following the commercial manufacturer (cat.# 78833; Thermo Fisher Scientific). Blots were developed using enhanced chemiluminescence.

### Hematoxylin-Eosin Staining and Immunohistochemistry

Liver tissues fixed in 10% formalin and embedded in paraffin were sectioned 4 μm in thickness and assessed for histologic changes by hematoxylin-eosin staining and Sirius red staining. IHC was performed using the EXPOSE Mouse and Rabbit Specific HRP/DAB Detection IHC kit and Rabbit anti-mouse PAI-1 antibody were purchased from Abcam (cat.# ab80436; cat.# ab182973). After hematoxylin-eosin staining or IHC, samples were observed under an optical microscope to determine the morphologic changes in liver and expression of PAI-1.

### TUNEL Assay

Apoptotic nuclei were detected in formalin-fixed liver tissue sections by the TUNEL staining using in situ apoptosis detection kit (cat.# ab206386; Abcam).

### Plasma PAI-1 and Aminotransferase Levels

PAI-1 concentration in mouse plasma was measured using a sandwich enzyme-linked immunosorbent assay (cat.# ab108891; Abcam) following the manufacturer’s instructions. Briefly, in microplates, standards (50 μL), samples (10 μL sample liquid and 40 μL diluent), and blank were set into predefined wells. In the wells for standards and samples, horseradish peroxidase-labelled conjugates (100 μL) were added before sealing the plates for incubation at 37°C for 1 hour. The plates were washed 5 times and then substrates A (50 μL) and B (50 μL) were added to each well. Following incubation at 37°C for 15 minutes, stop solution (50 μL) was added to each well and the absorbance of each well was measured at 450 nm.

For aminotransferases, blood from mice was collected and the plasma was separated by centrifugation (2000 *g* for 10 minutes at 4°C). Plasmatic levels of alanine aminotransferase and aspartate aminotransferase activities were measured using alanine aminotransferase and aspartate aminotransferase assay kit (Sigma-Aldrich) according to the manufacturer’s instructions.

### In Vivo Caspase-3 Activity Assay

Caspase-3 activity was determined using colorimetric assay kit, which used synthetic tetrapeptides (Asp-Glu-Val-Asp [DEAD] for caspase-3; pNA, cat.# A1086; Sigma-Aldrich, Merck KGaA) labeled with p-nitroaniline (cat.# ab39401; Abcam). The kit was used according to the manufacturer's protocols. Briefly, mouse livers were lysed in the supplied lysis buffer for 30 minutes at 4°C. Supernatants were collected and incubated with the supplied reaction buffer containing dithiothreitol and DEAD-pNA as substrates at 37°C. The reactions were measured by changes in absorbance at 405 nm using the FLUOstar Omega Microplate Reader (BMG Labtech, Cari, NC).

### PAI-1 Promoter Reporter Assay

Human *SERPINE 1* and murine *Serpine 1* promoter-luciferase reporter (SwitchGear Genomics, Menlo Park, CA) and p65 plasmids or empty vector (Genecopoeia, Rockville, MD) were cotransfected into primary human and mouse hepatocytes (0.4 × 10^6^ cells/well, 6-well plates) using JetPRIME reagent (New York, NY). After 48 hours of transfection, Cypridina and Renilla luciferase activities were measured using the LightSwitch Dual Assay System (SwitchGear Genomics) on a GloMax 20/20 Luminometer (Promega, Madison, WI). Promoter activity of Renilla luciferase activity was normalized by the Cypridina luciferase activity.

### Chromatin Immunoprecipitation Assay

Chromatin immunoprecipitation assays were performed using the *EZ-ChIP* kit (Millipore, Billerica, MA). Sonicated chromatin from mouse livers was immunoprecipitated with 3 μg of antibody against (cat.# ab16502; Abcam), reverse cross-linked and polymerase chain reaction amplified for 35 cycles with the following forward (Fw) and reverse (Rev) murine *Serpine 1* promoter primer sequences: Fw, 5’- TTTCTGTGGTAACCTCTGT-3’; Rev, 5’- ATGAAATGTGCCCTGTGAT-3’.

### Fluorescence Microscopy

To study the subcellular localization of NF-κB p65 subunit, hepatocytes were plated at a density of 0.5 x 10^5^ cells in 4-well chamber slide and pretreated with PureCol collagen I (Advanced BioMatrix, Carlsbad, CA). Cells were fixed, made permeable and processed for the direct immunofluorescence microscopy by the ICC Abcam protocol (Cambridge, MA) using anti-p65 (1:100, #16502; Abcam) and secondary antibodies (1:200, Alexa Fluor 594 goat anti-rabbit, cat.# A-11012; Thermo Fisher Scientific).

Mouse livers were fixed with 4% paraformaldehyde, paraffin-embedded, and stained against NF-κB p65 subunit or F4/80 or CD31. Briefly, after antigen retrieval (cat.# ab208572; Abcam) and permeabilization (0.5% Triton X), 4-μm sections were blocked with horse serum (cat.# 16050130; Thermo Fisher Scientific) for 30 minutes. Sections were subsequently incubated with anti-p65 (1:100, #16502; Abcam) or F4/80 antibody (1:100, Cat.# MF48000; Invitrogen) or anti-CD31 (1:100, cat.# NBP2-33154; Novus) at 4°C overnight. The following day, sections were incubated with secondary antibody (1:200: Alexa Fluor 488 donkey anti-rabbit, cat.# A-21206 [p65 and F4/80 sections]; Alexa Fluor 594 goat anti-mouse, cat.# A-11032 [CD31 sections]; Thermo Fisher Scientific) for 1 hour at room temperature. Finally, stained cells and sections were mounted using Vectashield mounting media with DAPI (cat.# H-1300-10; Vector laboratories, Inc, Burlingame, CA) and imaged with the Keyence BZX-700 microscope.

### Exosome Isolation and Incubation with KCs and LSECs

Exosomes from cell culture supernatants were isolated using ExoQuick-TC kit (System Biosciences) following the manufacturers' instructions. Briefly, hepatocyte-conditioned medium (10 mL) was collected after 24 hours and clarified by centrifugation at 12,000 × *g* for 25 minutes to remove any cell debris and possible apoptotic bodies. The supernatants were then incubated overnight with ExoQuick-TC exosome precipitation solution (cat.# EXOTC50A-1; System Biosciences) at 4°C and centrifuged at 1500 × *g* for 30 minutes to harvest the exosome pellet. The exosomes were resuspended in 100 μL 1 × phosphate-buffered saline and purity was verified with Western blot for exosomes markers, such as CD9 and heat shock protein 70. Lastly, 30 μL of exosomes were added to 1 mL of culture media of KCs and LSECs for 24 hours. The remaining supernatant was dialyzed using Slide-A-Lyzer Dialysis Cassettes (Thermo Fisher) and used to determine the level of the free form of PAI-1 by Western blot.

### PAI-1 Neutralization

Hepatocytes were treated with recombinant IL-1β (10 ng/mL) and supplemented with neutralizing antibody against PAI-1 (25 μg/mL)^58^ from Genetex (cat.# gtx89568) for 24 hours. Cells were washed twice with 1X phosphate-buffered saline and processed for proteins extraction.

### Detection of Lysosomal Activity and Glycolysis

Lysosomal acidification (activity) and glycolysis (L-lactate) levels in mouse hepatocytes were evaluated with Lyso-Tracker red staining (cat.# L7528; Thermo Fisher) and glycolysis assay kit (cat.# MAK439; Millipore Sigma), respectively, based on the manufacturers' instructions. The fluorescence intensity was observed using the Keyence BZX-700 microscope.

### Statistical Analysis

Data are expressed as mean ± standard error of the mean. Statistical analysis was performed using analysis of variance and Fisher test. For mRNA and protein levels, ratios of genes and proteins to respective housekeeping densitometric values were compared. Significance was defined by *P* < .05.
